# Myoprotective Potential of Creatine Is Greater than Whey Protein after Chemically-Induced Damage in Rat Skeletal Muscle

**DOI:** 10.3390/nu10050553

**Published:** 2018-04-30

**Authors:** Matthew B. Cooke, Emma Rybalka, Christos G. Stathis, Alan Hayes

**Affiliations:** 1 Department of Health and Medical Sciences, Swinburne University of Technology, Hawthorn, VIC 3122, Australia; 2 Institute for Health and Sport, Victoria University, Melbourne, VIC 3000, Australia; Emma.Rybalka@vu.edu.au (E.R.); christos.stathis@vu.edu.au (C.G.S.); alan.hayes@vu.edu.au (A.H.); 3 Australian Institute for Musculoskeletal Science (AIMSS), Victoria University, Melbourne, VIC 3021, Australia; 4 Department of Medicine-Western Health, The University of Melbourne, 176 Furlong Road, St Albans, VIC 3021, Australia

**Keywords:** dietary supplementation, injury, regeneration

## Abstract

The myoprotective effects of creatine monohydrate (CR) and whey protein (WP) are equivocal, with the use of proxy measures of muscle damage making interpretation of their effectiveness limited. The purpose of the study was to determine the effects of CR and WP supplementation on muscle damage and recovery following controlled, chemically-induced muscle damage. Degeneration of the extensor digitorum longus (EDL) muscle was induced by bupivacaine in rats supplemented with either CR, WP, or standard rat chow (CON). At day 7 and 14 post-myotoxic injury, injured EDL muscles were surgically removed and tested for isometric contractile properties, followed by the contralateral, non-injured EDL muscle. At the completion of testing, muscles were snap-frozen in liquid nitrogen and stored for later analysis. Data were analyzed using analysis of variance. Creatine-supplemented muscles displayed a greater proportion of non-damaged (intact) fibers (*p* = 0.002) and larger cross-sectional areas of regenerating and non-damaged fibers (*p* = 0.024) compared to CON muscles at day 7 post-injury. At day 14 post-injury, CR-supplemented muscles generated higher absolute forces concomitant with greater contractile protein levels compared to CON (*p* = 0.001, *p* = 0.008) and WP-supplemented muscles (*p* = 0.003, *p* = 0.006). Creatine supplementation appears to offer an element of myoprotection which was not observed following whey protein supplementation.

## 1. Introduction

The etiology of the damage/repair process and alterations in muscle function following unaccustomed, eccentric exercise has been extensively studied [1,2,3,4]. Disturbances in the regulation and concentration of intracellular Ca^2+^ and changes in the rate of muscle protein degradation appear to be an integral part of this process [5,6,7]. Enhancing muscle recovery could involve ameliorating the initial insult of injury by improving the Ca^2+^ handling ability of the muscle and/or increasing the rate of regeneration by augmenting muscle protein synthesis. While other contributing factors, such as post-injury inflammation, and proliferation and differentiation rates of muscle satellite cells and myoblasts, respectively, can modulate regenerative capacity, the ability of muscle to buffer the initial physiological damage signal and induce regenerative activity remain fundamental in achieving complete functional recovery [4].

Dietary supplements creatine monohydrate (CR) and whey protein (WP) have previously demonstrated ergogenic roles in exercise performance and recovery, and subsequent adaptations from exercise training [8,9]. However, the effects of CR and WP supplementation on indicators of muscle damage and recovery after injury in both human [10,11,12,13,14,15,16,17,18,19,20,21,22,23,24,25] and animal [26,27,28] models are equivocal. Studies using acute ingestion of CR before (~20 g/day) and/or after exercise (~2–3 g/day) have reported no effect on the extent of muscle damage and/or recovery following a high-force, eccentric exercise [12] or low force, hypoxic resistance exercise challenge [11], respectively. Similarly, but utilizing a higher dosage of 40 g/day before and 10 g/day after exercise, CR ingestion had no significant benefit on markers of muscle damage and recovery following eccentric contractions of the elbow flexor muscles [13]. These observations have also been supported following chemically-induced muscle damage, with CR supplementation ineffective in accelerating the time course of muscle recovery in rodents [27]. In contrast, Bassit et al. [26] showed that acute CR supplementation (5 g kg^−1^ body weight per day) for five days was able to attenuate muscle strength loss and rise in plasma markers of muscle damage (i.e., lactate dehydrogenase (LDH) and creatine kinase (CK)) following electrically-induced muscle damage in rats. Furthermore, the same authors reported potential protective effects in triathletes following an ironman competition, albeit, with a low sample size [26]. In support of these findings, Cooke et al. [10] reported higher isometric and isokinetic leg extension strength and lower plasma CK levels when CR was supplemented before (~20 g/day) and during the days after (~7 g/day) an intense resistance exercise session.

The majority of WP supplementation studies have focused on the purported benefits of higher protein intake during periods following damaging exercise to enhance recovery [9]. White and colleagues [16] found no significant improvement in the rate of muscle recovery following maximal isokinetic eccentric contractions of the quadriceps when WP (23 g) was ingested pre- or post-exercise. Conversely, Buckley et al. [23] showed improved muscle strength recovery when WP hydrolysate (25 g) was ingested during the days following an intense exercise bout (i.e., 100 maximal knee extensions of the knee extensors) compared to a placebo. However, given no significant elevation in an indirect marker of muscle damage (i.e., CK) was observed, reduced fatigue, rather than attenuation of muscle damage most likely occurred. Notwithstanding, consumption of WP isolate (~25–30 g, 4 times a day) during the days following an intense resistance exercise session attenuated decrements in muscle strength and rises in LDH [25].

A limitation in human studies is the use of proxy measures of muscle damage/recovery. This can introduce confusion regarding whether the benefits of supplementation are beyond the initial fatigue recovery or the result inherent to damage. Moreover, the damage protocols typically used in human studies (i.e., downhill running, isolated eccentric contractions) result in high variability in the magnitude of muscle damage, and consequently the effects from the supplement intervention are often variable. Thus, the use of animal models allows for a more direct and comprehensive analysis of muscle damage and regeneration following injury. The purpose of this study was to examine temporal changes in functional, morphological, and biochemical characteristics of muscle recovery following a controlled, standardized, chemically-induced injury to identify potential myotherapeutic benefits of CR and WP. We hypothesized that CR and WP supplementation would restore injury-induced loss of isometric contractile strength sooner by either blunting the extent of the initial damage and/or improving the rate of fiber regeneration.

## 2. Materials and Methods

### 2.1. Animals

Fifty-one male Sprague–Dawley rats (*Rattus norvegicus*) weighing 212.4 + 25.1 (mean ± standard deviation (SD)) were randomly separated into three groups: (i) standard rat chow (CON) *n* = 18; (ii) CR-supplemented *n* = 15; and (iii) WP-supplemented *n* = 18. Five animals (three CON and two WP) were not used for subsequent contractile and/or biochemistry and histological analysis due to the following reasons: animal died under anaesthesia (two animals); and isolated muscles dissected could not be tested due to surgical procedure issues (three animals). Control rats were fed standard rat chow for a period of 14 days prior to, and 14 days following chemically-induced damage. CR-supplemented rats were fed CR monohydrate (AST Sports Science, CO, USA) as a 0.02% (2 g creatine/100 g standard rat chow) mixture for five days (loading phase), and a maintenance dosage of 0.002% (0.2 g creatine/100 g standard rat chow) mixture for nine days prior to chemically-induced damage. Following injury, rats continued to consume the maintenance dosage (0.002%) for either 7 or 14 days. WP-supplemented rats were fed WP isolate (AST Sports Science, CO, USA) at 5 g kg^−1^ body weight per day in the chow for a period of 14 days following chemically-induced damage. The CR and WP dosages used in the current study were converted from a human equivalent dose on the basis of body surface area [29]. The human equivalent dose for CR was 0.3 g kg^−1^ body weight per day for the loading phase and 0.03 g kg^−1^ body weight per day for the maintenance phase [8]. The human equivalent dose for WP was ~0.25 g kg^−1^ body weight every four hours over a 24 hour period [30]. Since WP was provided in the chow and not given as a single bolus every four hours, we administered ~60 g of WP per day based on rats consuming ~20 g every four hours over a 12-hour period (dark cycle). For a 70 kg individual, this would equate to ~0.85 g kg^−1^ body weight per day, or ~5.27 g kg^−1^ body weight per day for rats (rounded down to 5 g for ease of supplement mixing). Animals were acquired from Monash Animal Services (Monash University, Melbourne, Australia) and housed at the animal holding facility at Werribee campus, Victoria University. All procedures described below received ethical approval from the Victoria University Animal Ethics Committee and conformed to the “Principles of laboratory animal care” (NIH publication No. 86–23, revised 1985) and the Australian Code of Practice for the Care and Use of Animals for Scientific Purposes. Animals were housed at a constant temperature (22 °C) under a 12:12-h light-dark photoperiod. Animals were housed in pairs (body weight matched) prior to muscle injury in accordance with ethical approval. While a more accurate measurement of the level of CR and WP supplementation would be obtained if the rats were housed separately, there was no evidence to suggest that there was an uneven distribution of supplementation between cage mates, since rats were fed ad libitum, and similar increases in body mass were seen in both rats in each cage. All animals were housed separately following muscle injury in accordance with ethical approval, with access to standard or supplemented chow ad libitum.

### 2.2. Experimental Procedures

All rats were lightly anesthetized with a Domitor (10 mg kg^−1^ body weight) and ketamine (6 mg kg^−1^ body weight) combination via an intraperitoneal injection (i.p.), such that they were unresponsive to tactile stimuli. The fur on the hindlimb was cut short, shaved, and cleaned using standard aseptic techniques. The extensor digitorum longus (EDL) muscle was then surgically exposed, with care taken to avoid damaging its nerve and blood supplies, and then injected with 0.5% bupivacaine hydrochloride (bupivacaine) (Bupivacaine Hydrochloride, Pharmacia and Upjoin (Perth) Pty. Ltd., Bentley, WA, Australia), through several injections in the distal, proximal, and mid-belly regions of the muscle, using a 26-gauge needle. All muscles received the same amount of bupivacaine (0.5 mL). This procedure causes degeneration of most fibers in the injected muscle [31]. The contralateral EDL muscle served as an uninjured control, and was not injected intramuscularly as previous research has shown that little or no damage is caused by the insertion of the needle itself, or the injection of an otherwise harmless vehicle such as isotonic saline [31]. Following injury, the small incision was closed with Michel suture clips and swabbed with Betadine antiseptic (povidone iodine solution). Anaesthesia was reversed using Antisedan® (Atipamezole Hydrochloride, Zoetis Inc. Parsippany, NJ, USA) and animals continued their dietary modification.

### 2.3. Assessment of Contractile Properties

At 7 and 14 days post-injury, rats received intra-peritoneal injections of 60 mg kg^−1^ body weight Nembutal. A longitudinal incision was made into the injured hind leg of the rat. Each muscle was carefully dissected free from other tissues, starting from the proximal end, and placed into a horizontal, custom-built plexiglass bath containing a Krebs–Henseleit Ringer solution (NaCl 118 mM; KCl 4.75 mM; Na_2_HPO_4_ 1 mM; MgSO_4_∙7H_2_O 1.18 mM; NaHCO_3_ 24.8 mM; CaCl_2_ 2.5 mM; and d-Glucose 11.0 mM). The buffer solution was maintained at a pH of 7.4 and temperature of 25 °C. For all muscles, optimal muscle length (Lo), force-frequency relationship, peak twitch force (Pt), and maximum isometric force (Po) were determined according to previously published procedures from our lab [32]. Optimal fiber length (Lf) was determined with the previously established Lf-to-Lo ratio of 0.44 for the EDL muscle [33]. Peak tetanic forces were expressed relative to the muscle’s cross-sectional area (specific force (sPo)) enabling comparisons between muscles of different area and length. Muscle cross-sectional area (CSA) was calculated taking into account muscle mass, Lf, and 1.06 g/m^3^, the density of mammalian skeletal muscle [34]. Immediately after the functional measurements, the muscle was removed from the bath, blotted dry on filter paper, and weighed. The muscle was then divided into two sections; one-half was coated with optimal cutting temperature (OCT) compound and snap-frozen in isopentane, cooled in liquid nitrogen, and stored for subsequent histological analysis. The other portion was immediately snap-frozen and stored for subsequent muscle protein analysis. The same procedure was then performed on the contralateral, non-damaged EDL muscle. Following removal of all muscles required for analysis, rats were killed by overdose of anaesthetic.

### 2.4. Histological Analysis

Histological procedures were performed to examine the effects of dietary supplementation on general tissue morphology of both injured and uninjured muscles, following 7 and 14 days of treatment. Using a cryostat microtome set at −20 °C (Microm GmbH D-6900; Heidelberg, West Germany), transverse sections (10 µm thick) were cut from each muscle sample as close to the mid-belly as possible, and placed onto a microscope slide. Sections were fixed in 10% buffered formol saline solution and stained with routine haematoxylin and eosin, as previously described [35]. Digital images of muscle sections were obtained using a camera (Zeiss Axiolab; Carl Zeiss GmbH, Jena, Germany) attached to an upright Carl Zeiss light microscope (Zeiss Axiolab; Carl Zeiss GmbH, Jena, Germany) at 5×, 10×, and 20× magnification. Each section was examined in a single blinded manner, using calibrated Analytical Imaging Station (AIS, v6.0 Imaging Research, Ontario, Canada) software. The mean cross sectional (CSA) of individual fibers was calculated by interactive determination of the circumference of at least 150 adjacent fibers from the center of each muscle section [36]. A quantitative estimate of the relative affected area within cross sections of muscle samples was determined using a similar method described elsewhere [37]. Each muscle section was categorized into three distinct regions: (1) muscle damage (i.e., necrosis, inflammatory cell accumulation, and few small-multinucleated regenerating fibers); (2) regenerating fibers (i.e., muscles showing larger regenerating fibers with centrally located nuclei); and (3) normal fibers (i.e., muscles showing multi-nucleated intact fibers).

### 2.5. Protein Analysis

The muscle protein content was assessed in both injured and uninjured muscles, following 7 and 14 days of treatment, according to a modified version of methods described by Beitzel et al. [38]. Briefly, approximately 10–20 mg of the muscle were ground-glass homogenized on ice in 50 µL of ice-cold, homogenizing buffer solution A (containing in mM: KCl 50, KH_2_PO_4_ 10, MgCl_2_∙6H_2_O 2, EDTA 0.5, DTT 2) per mg of muscle (i.e., a 1:50 wet weight: volume dilution). Two hundred microliters of crude homogenate were transferred into a labelled cryule and immediately snap-frozen and stored in liquid nitrogen for later assessment of total protein concentration. The remainder of the homogenate was centrifuged at 5 °C for 10 min at 1000 G. The supernatant, containing the cytosolic proteins, was discarded and the pellet, containing the contractile proteins, was re-suspended in 200 µL of the ice-cold, homogenizing buffer solution A. The suspension was transferred into a labelled cryule and immediately snap-frozen and stored in liquid nitrogen for later assessment of contractile protein concentration. Protein concentration was determined according to methods described by Bradford [39] using a Bradford Protein Assay (Bio-Rad Protein assay, Bio-rad Laboratories, Hercules, CA, USA).

### 2.6. Statistical Analysis

Data normality was assessed by Shapiro–Wilk test. All values are reported as means ± standard deviation (SD). Statistical evaluation for each recovery time point (i.e., day 7 and day 14) was accomplished using a two-way analysis of variance (ANOVA) with supplement groups (CR, WP, and CON), and experimental condition injured and uninjured (NORM) as factors. When appropriate, differences between groups were tested with a Newman–Keuls post-hoc test, especially to compare values measured during muscle regeneration with those observed in contralateral uninjured muscle. Differences in animal morphology characteristics between groups were assessed by unpaired (independent) students’*t*-test. Target and observed supplementation dosage within groups was assessed by chi-square test. An alpha value 0.05 was considered statistically significant.

## 3. Results

### 3.1. Supplement Ingestion

Creatine-supplemented rats consumed 318 ± 18 mg per day for five days and 29 ± 3 mg per day for nine days prior to myotoxic injury. These supplement intakes were lower than the expected target dosage of 475 mg and 50 mg per day, respectively. Following myotoxic injury, CR-supplemented rats consumed 28 ± 5 mg and 31 ± 0.5 mg per day, for 7 and 14 days, respectively. These observed CR supplement intakes were also lower than the expected target dosage of 50 mg per day. Rats supplemented with WP for 7 days and 14 days following myotoxic injury consumed 699 ± 10 mg and 724 ± 9 mg per day, respectively. Similar to the CR-supplemented rats, the WP intake was lower than the expected target dosage of 1.25 g per day following myotoxic injury, and thus, the target dosages for both supplements were not achieved post-injury/surgery.

### 3.2. Body Weight and Muscle Mass

Body weights for all groups are presented in Table 1. When expressed as a change from initial body weight, CR-supplemented rats gained significantly more body mass (approximately 6 g) compared to CON after 2-weeks supplementation (Group 1, *p* < 0.05). A similar trend was observed for CR-supplemented rats in Group 2, although this was not deemed statistically significant (*p* = 0.094). No significant changes were observed in the WP-supplemented animals when expressed relative to initial body weight (Table 1).

The effects of bupivacaine-induced myotoxicity on contractile, histological, and protein properties in muscle have been described extensively elsewhere [40,41]. Thus, only the effects of the investigational supplements are described in this paper. However, where appropriate, statistical differences between injured and contralateral muscles are reported in tables and figures. In addition, CR-supplemented muscles and WP-supplemented muscles are further categorized as CR-INJ and CR-NORM, and WP-INJ and WP-NORM for injured and uninjured muscles, respectively, while CON muscles are categorized as CON-INJ and CON-NORM for injured and uninjured muscles, respectively.

Muscle mass changes in CON-, CR-, and WP-supplemented EDL muscles at day 7 and 14 post-injury are summarized in Table 2. No significant differences in muscle mass were evident between groups at day 7 post-injury (*p* < 0.05). A significant group effect was observed at day 14 post-injury (*p* = 0.011), with subsequent analysis indicating CR-supplemented muscles were significantly heavier than WP–supplemented (*p* = 0.003) muscles in both injured and uninjured muscles, with only a trend observed against CON (*p* = 0.089). No differences in muscle mass to body mass ratio (MM:BM) were evident between all groups at day 7 post-injury. However, by day 14 post-injury, a significant group effect was observed (*p* = 0.025), with subsequent analysis revealing greater MM:BM in the CR-supplemented animals compared to CON (*p* = 0.044) and WP-supplemented (*p* = 0.010) animals. No group by experiment interactions were observed for muscle mass or MM:BM (Table 2).

### 3.3. Contractile Properties

**Figure 1:**
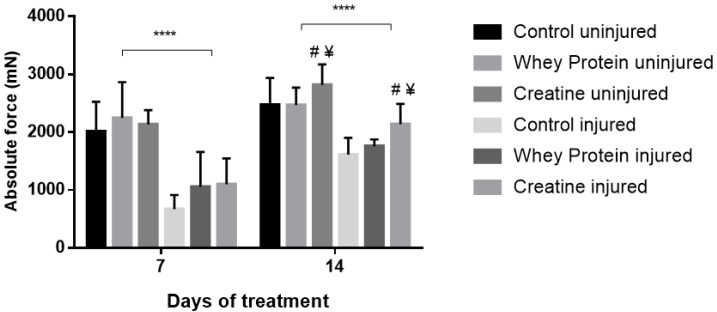
Absolute forces (Po) of uninjured and injured EDL muscles from rats treated with standard rat chow- (CON-), whey protein- (WP-), or creatine- (CR-) supplemented rat chow at day 7 and 14 post-injury. Values are mean ± SD. **** (*p* < 0.0001) significantly different from contralateral values. # (*p* < 0.05) significantly different from CON muscles at day 7 and/or day 14 post-injury. ¥ (*p* < 0.05) significantly different from WP-supplemented muscles at day 7 and/or day 14 post-injury.

**Figure 2:**
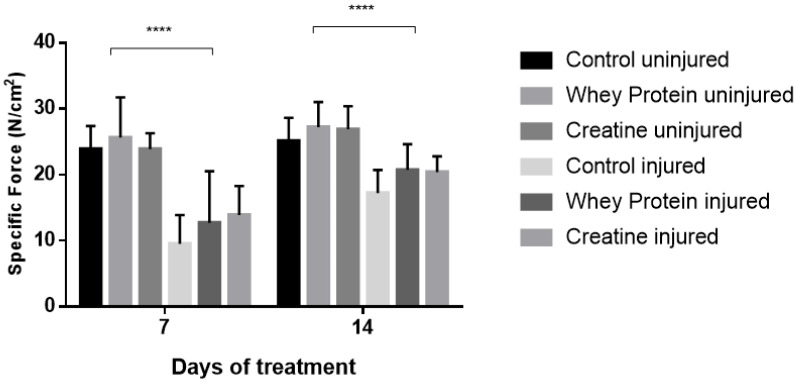
Specific forces (sPo) of uninjured and injured EDL muscles from rats treated with standard rat chow- (CON-), whey protein- (WP-), or creatine- (CR-) supplemented rat chow at day 7 and 14 post-injury. Values are mean ± SD. **** (*p* < 0.0001) significantly different from contralateral values.

Contractile properties of all muscles are summarized in Table 2. CR and WP supplementation had no significant effect on optimal length (Lo) at day 7 (*p* = 0.361) and day 14 (*p* = 0.802) post-injury. Conversely, a significant group effect was observed for half relaxation time (½RT) at day 7 (*p* = 0.024), with CR-supplemented muscles demonstrating significantly faster ½RT compared to CON (*p* = 0.011) and WP-supplemented muscles (*p* = 0.026) post-injury. This was also evident at day 14, with significantly faster ½RT in the CR-supplemented and WP-supplemented muscles compared to CON (*p* = 0.011, *p* = 0.031, respectively). CR and WP supplementation had no significant effect on time to peak tension (TTPT) at day 7 or day 14 (*p* < 0.05) post-injury. Similarly, there were no differences in peak twitch force (Pt) at day 7 post-injury (*p* < 0.05). Conversely at day 14 post-injury, a main group effect was observed for Pt (*p* = 0.009), with significantly higher Pt observed in the CR-supplemented compared to WP-supplemented muscles (*p* = 0.003). A strong trend towards significance was evident when compared to CON muscles (*p* = 0.057). A significant main group effect was observed for absolute force (Po) at day 14 (*p* = 0.001), but not day 7 post-injury (Figure 1). Subsequent analysis revealed that CR-INJ and CR-NORM muscles generated significantly higher forces than CON-INJ and CON-NORM (*p* = 0.001), and WP-INJ and WP-NORM muscles (*p* = 0.003, Figure 1). When Po was normalized for muscle size, a main group effect trend for specific forces (sPo) was observed at day 14 post-injury (*p* = 0.071, Figure 2), with no significant differences at day 7 post-injury (Figure 2).

### 3.4. Total and Contractile Protein

Muscle protein content was measured to confirm whether alterations in muscle mass could be observed at the ultra-structural level (Table 3). Following 7 days recovery, no significant differences between groups were observed for total protein and contractile protein levels (*p* > 0.05). By day 14 post-injury, no significant differences between groups for total protein were evident; however, a significant main group effect was detected for contractile protein content (*p* = 0.008). Significantly higher contractile protein content was noted between CR-INJ and CR-NORM compared to CON-INJ and CON-NORM muscles, respectively (*p* = 0.008), and WP-INJ and WP-NORM muscles, respectively (*p* = 0.006). No significant differences in total protein and contractile protein were noted between WP and CON muscles. In addition, CR and WP supplementation had no significant effects on contractile protein percentage at any time point relative to the CON.

### 3.5. Histological Analysis

**Figure 3:**
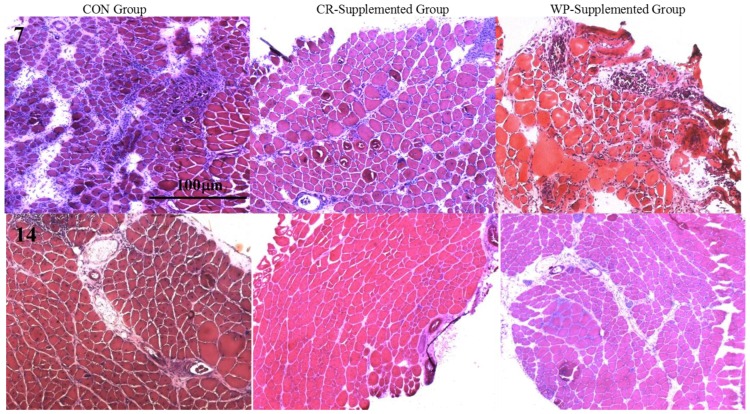
Hematoxylin and eosin-stained sections of injured EDL muscles from rats treated with standard rat chow- (CON-), whey protein- (WP-), or creatine- (CR-) supplemented rat chow at 7 and 14 days post-injury. Scale applies to all panels.

**Figure 4:**
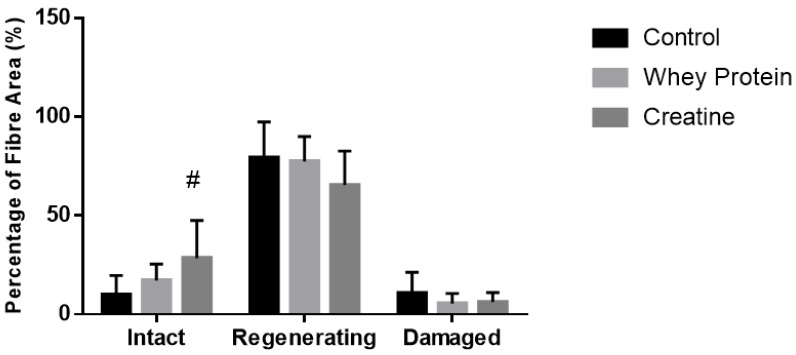
Muscle fiber recovery (expressed as a % of muscle CSA) of injured EDL muscles from rats treated with standard rat chow- (CON-), whey protein- (WP-), or creatine- (CR-) supplemented rat chow at day 7 post-injury. Values are mean ± SD expressed as either % of damaged fibers, regenerating fibers, and undamaged (intact) fibers. # (*p* < 0.01) significantly different from the injured CON values.

**Figure 5:**
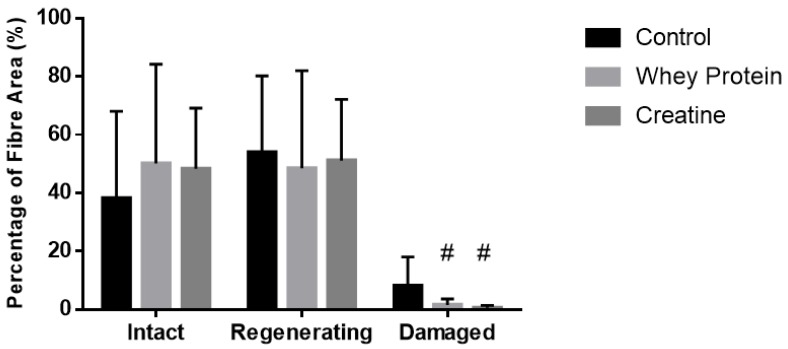
Muscle fiber recovery (expressed as a % of muscle CSA) of injured EDL muscles from rats treated with standard rat chow- (CON-), whey protein- (WP-), or creatine- (CR-) supplemented rat chow at day 14 post-injury. Values are mean ± SD expressed as either % of undamaged (intact) fibers, regenerating fibers, and damaged fibers. # (*p* < 0.01) significantly different from the injured CON values.

Cross-sectional area of both intact (non-damaged) and regenerating muscles was measured to determine whether the observed increases in muscle strength were due to changes in fiber hypertrophy (Table 2). At day 7 post-injury, a significant main group effect was observed (*p* = 0.032), with subsequent analysis revealing that the CSA of the regenerating and intact fibers of the CR-supplemented muscles was significantly larger than the CON muscles (*p* = 0.010). No effects of supplementation were evident at day 14 post-injury. Quantitative analysis (See Figure 3) of three distinct regions (i.e., muscle damage, regenerating fibers, and normal fibers) showed a greater percentage (~19%) of non-damaged fibers at day 7 post-injury following CR supplementation compared to CON (*p* = 0.002, Figure 4). By day 14, less damage was noted between CR-supplemented animals compared to CON (*p* = 0.024), and WP-supplemented animals compared to CON (*p* = 0.039, Figure 5).

## 4. Discussion

Creatine supplementation prior to and following a controlled myotoxic injury episode was more effective at restoring functional strength compared to whey protein and placebo supplementation. This enhanced functional capacity was associated with reduced damage following the initial insult, greater CSA of regenerating fibers during the early stages of recovery, and higher levels of muscle contractile protein content during the later stages of recovery. On the contrary, WP seemed to have delayed effects on muscle recovery, both functionally and structurally, following myotoxic injury. The findings from the present study provide important information on the morphological and biochemical mechanisms by which CR and WP are influencing recovery and elucidate the mechanisms for improvements observed in human studies using similar supplementation protocols.

Numerous studies have confirmed that supplementation with CR in conjunction with programmed resistance training is effective for augmenting gains in body and fat-free mass, and muscular strength in both men and women [42,43,44,45]. These benefits are possibly due to enhanced training-induced increases in satellite cell number and myonuclei concentration within skeletal muscle fibers [46]. Furthermore, CR supplementation alone may increase muscle mass, which is likely a reflection of increased water retention, rather than effects on mixed muscle protein synthesis [47,48]. In the present study, animals consuming CR gained more body weight (approximately 6 g) than animals consuming standard rat chow over a two-week loading period, with this trend continuing during the 14 days post-surgery period, but to a lesser degree. By day 14 post-injury, both injured and uninjured muscles of the CR-supplemented animals were heavier than WP and CON muscles. The heavier injured muscles of the CR-supplemented animals could be due to faster recovery as evidenced by larger CSA of the regenerating fibers and higher contractile protein levels. Indeed, cell culture studies have shown accelerated differentiation of myoblasts into hypertrophic myotubes as a result of creatine’s ability to offset the inhibition of myogenic differentiation typically caused by high levels of oxidative stress following injury [49,50,51]. The uninjured muscles also displayed higher total protein levels and greater fiber CSA, suggesting CR-induced anabolic growth driven by increased cellular hydration status [52]. In contrast, WP-supplemented animals displayed similar weight gain compared to the CON animals, and no significant changes in fiber CSA and protein levels.

A primary outcome of this study was to determine whether CR and WP supplementation could enhance the functional recovery of the muscle following myotoxic injury. Absolute forces in the injured muscles of the CR-supplemented animals were closer to full recovery (~76% of uninjured values) compared to injured WP muscles (~71% of uninjured values) and CON muscles (~65% of uninjured values) by day 14 post-injury. Furthermore, both injured and uninjured muscles following CR supplementation generated significantly higher forces than WP- and CON-supplemented muscles. However, when corrections were made for cross-sectional area, specific forces were not significantly different between groups. It should be noted that specific force, an indicator of force output based on a given muscle size, can be influenced during the early stages of muscle regeneration by inflammation and muscle swelling, and thus, may explain the non-significant differences between groups.

Histological analysis revealed a significantly higher proportion of intact (non-damaged) fibers in the regenerating CR muscles compared to CON muscles during the early stages post-injury. In addition, CSA of the regenerating and intact fibers was significantly larger in the CR-supplemented muscles compared to the CON muscles. These observations suggest that CR supplementation is reducing the extent of damage and/or enhancing the growth of the regenerating fibers. Though no corresponding functional enhancement was noted at this time point (i.e., day 7) for CR supplementation, it appears that morphological improvements occurring at this early stage may underpin the benefits observed in the later stages of recovery (i.e., higher absolute forces at day 14).

Originally considered solely as a sports supplement, creatine has shown over the past two decades that its role goes beyond cellular bioenergetics, with pleiotropic effects that converge to maintain cell homeostasis, protect membranes, and reduce oxidative stress and apoptosis [50,53,54]. The reduced magnitude of damage following the initial injury insult could be due to improved calcium (Ca^2+^) handling ability of the muscle, and thus, less activation of self-accelerating, degradative pathways that lead to damage and degeneration [55]. CK isoforms, together with its substrates CR and phosphocreatine (PCr), represent an intricate cellular energy buffering and transport system [56]. The sarcoendoplasmic reticulum (SR) Ca^2+^ transport ATPase (SERCA) pump derives its adenosine triphosphate (ATP) preferentially from PCr via SR-bound CK. A high ATP/adenosine diphosphate (ADP) ratio within the vicinity of the SERCA pump allows the pump to function optimally [57]. During times of severe stress such as post-injury, increased PCr stores following CR supplementation may enhance the function of these pumps, and thus, the Ca^2+^ handling of the muscle. Furthermore, the antioxidant properties of CR may reduce the reactive oxygen species (ROS)-induced inhibition of SERCA pump function [58], as well as damage to RNA and inhibition of mitochondrial permeability transition, an early event in apoptosis [59,60].

Although SERCA pump activity was not measured directly in the current study, increased Ca^2+^ uptake rate by SR vesicles from tibialis anterior muscle following CR supplementation has been shown in a previous study [61]. In the present study, the rate of muscle relaxation, which is an indirect measurement of Ca^2+^ handling ability of the muscle, was significantly increased in both the injured and uninjured muscles at day 7 and 14 post-injury following CR supplementation. This indirect measurement of Ca^2+^ handling ability of the muscle could indicate CR supplementation is enhancing SERCA pump function. ROS levels were not measured in the current study, and therefore, we can only speculate on the role of ROS in the current study findings.

An interesting observation was that CR intake prior to injury and post-injury were lower than the expected target dosages. The conversion calculations to determine the target dosages for CR was based on an average rat weight of ~250 g. For example, given that CR-supplemented rats consumed on average 318 ± 18 mg during the loading phase, and weighed closer to ~200 g, their dosage per kg.bw would be around 1.6 g kg^−1^ body weight per day. If we divide by the rat conversion factor (6.2), we obtain a value of 0.256 g kg^−1^ body weight per day, which for a 70 kg individual would equate to ~18 g of CR per day. This is very close to the recommended dosage of ~20 g per day for loading. Despite the lower intakes, it was clear that the beneficial effects of CR supplementation were still apparent in the present study. Lower than expected target dosages were also seen in the WP-supplemented animals post-injury, with supplement intakes lower than the supplement target dosage of 5 g kg^−1^ body weight per day at day 7 and 14 post-injury. Given similar calculations as described above, our rats were consuming around ~33 g per day, which is lower than the targeted ~60 g per day, and could be contributing to the minimal benefits observed from WP.

A number of limitations exist in the current study. Firstly, intramuscular PCr and CR levels were not measured. Both injured and uninjured EDL muscles were tested for contractile properties, lasting approximately 40 minutes, before they were frozen. Thus, variation in PCr breakdown for each muscle would make it difficult to determine the effectiveness of the loading phase. Notwithstanding, given the changes observed in body weight, muscle mass, and force output, we can assume that levels within the muscles increased by ~20%, as typically reported following a standard loading regimen [62]. Secondly, myotoxic injury results in a greater magnitude of damage, specifically the extent of muscle necrosis, compared to human models of injury. This should be carefully considered when making comparisons between models. The processes of regeneration are very similar in all models, albeit the absolute values reported on the extent and trajectories of the regenerative process, which may vary considerably [63]. Finally, rats in the current study consumed their recommended dosage of WP in chow over a period of time rather than in one meal via oral gavage, as performed in other studies [64,65]. We chose this route of administration to avoid additional stress on the injured animals. However, we acknowledge that not delivering the WP in one meal/bolus, could be a limitation to our supplementation protocol.

## 5. Conclusions

In summary, reducing the magnitude of damage following the initial injury to the muscle led to enhanced functional and morphological characteristics during the later stages of muscle recovery, following creatine supplementation. In contrast, whey protein supplementation demonstrated less impact on the histological, functional, and morphological characteristics of muscle recovery following injury. The observed myoprotective effect of CR supplementation could be due to improved calcium handling ability of the muscle following the initial insult and during the early recovery period. To gain a better understanding of the regenerative processes occurring post-injury and the mechanisms behind the supplements, future work should explore markers of satellite cell activation (i.e., Pax7^+^) and expression of embryonic and neonatal myosin heavy chains. Furthermore, in vitro experiments with myogenic progenitor cells supplemented with CR and WP would also be insightful. Reducing the extent of initial damage and enhancing muscle recovery will not only benefit athletes during intense training phases, competition, and recovery from injury, but may also have important clinical implications in various myopathies.

## Figures and Tables

**Table 1:** The effect of standard rat chow diet (CON), creatine (CR), and whey protein (WP) supplementation on animal body weights (BW) (g).

**Group 1**	**Initial BW (g)**	**BW (g) after 14 Days**	**BW (g) after 21 Days**
CON (*n* = 9)	184.8 ± 23.6	209.4 ± 27.9	222.5 ± 24.2
CR (*n* = 7)	201.4 ± 16.4	232.3 ± 20.0 #	248 ± 18.5
**Group 2**	**Initial BW (g)**	**BW (g) after 14 days**	**BW (g) after 28 days**
CON (*n* = 9)	217.3 ± 15.7	243.7 ± 19.9	259.2 ± 21.4
CR (*n* = 8)	210.6 ± 14.8	242.0 ± 19.1	260.1 ± 19.5
**Group 3**	**Initial BW (g)**	**BW (g) after 7 days**	
WP (*n* = 9)	224.8 ± 24.8	234.4 ± 18.1	
**Group 4**	**Initial BW (g)**	**BW (g) after 14 days**	
WP (*n* = 9)	232.9 ± 22.3	249.1 ± 28.6	

Note: Values are presented as means ± SD. CR-supplemented groups were measured two weeks prior to injury (initial Body weight (BW)), on the day of injury protocol (BW after 14 days), and on either day 7 post-injury (Group 1: BW after 21 days) or day 14 post-injury (Group 2: BW after 28 days). Rat BW (g) for WP-supplemented groups were measured at day 7 (Group 3) and 14 (Group 4) post-injury. Please note that for Group 3 and 4, initial body weight is on the day of surgery; # (*p* < 0.05) significantly different from control muscles.

**Table 2:** Morphometric and contractile properties of uninjured and injured EDL muscles from rats treated with standard rat chow- (CON), whey protein- (WP), or creatine- (CR) supplemented rat chow at 7 and 14 days post-injury.

Treatment	CON-INJ	WP-INJ	CR-INJ	CON-NORM	WP-NORM	CR-NORM
Duration						
**7 days**						
*n*	8	8	7	8	8	7
MM (mg)	100.2 + 21.7 *	115.5 + 28.9 *	113.2 + 6.7 *	117.9 + 27.3	124.0 + 25.7	129.1 + 14.5
CSA (µm^2^)	740.9 ± 99 ***	948.2 ± 163 ***	1082.4 ± 80 ***#	2046.6 ±149	2087.9 ± 561	2353.8 ± 463 #
MM/BM (mg∙g^−1^)	0.44 + 0.07 *	0.50 + 0.10 *	0.49 + 0.05 *	0.54 + 0.09	0.52 + 0.08	0.54 + 0.06
Lo (cm)	2.9 ± 0.2	3.0 ± 0.2	2.9 ± 0.1	2.9 ± 0.1	3.0 ± 0.1	2.9 ± 0.1
Pt (mN)	120.1 ± 46 ***	278.1 ± 190 ***	220.7 ± 100 ***	610.6 ± 263	582.6 ± 238	493.9 ± 158
TTP (ms)	51.4 ± 11.9	54.6 ± 10.7	45.5 ± 13.3	46.0 ± 6.3	46.3 ± 13.7	44.8 ± 10.0
½ RT (ms)	82.1 ± 19.9 **	78.4 ± 25.9 **	53.3 ± 11.8 **¥#	57.4 ± 8.8	56.6 ± 20.1	51.4 ± 13.1 ¥#
**14 days**						
*n*	7	8	8	7	8	8
MM (mg)	139.2 + 21.7	124.6 + 25.7	150.6 + 12.0¥	145.4 + 11.8	139.4 + 20.3	153.9 + 8.8¥
CSA (µm^2^)	1476.5 ± 311 ***	1648.8 ± 205 ***	1627.6 ± 261 ***	2287.1 ± 222	2433.9 ± 385	2497.7 ± 319
MM/BM (mg∙g^−1^)	0.52 + 0.08	0.50 + 0.07	0.58 + 0.07¥#	0.56 + 0.04	0.56 + 0.07	0.60 + 0.04¥#
Lo (cm)	3.1 ± 0.1	3.1 ± 0.1	3.1 ± 0.1	3.2 ± 0.3	3.1 ± 0.1	3.1 ± 0.2
Pt (mN)	452.2 ± 57 ***	383.9 ± 133 ***	562.9 ± 69 ***¥	713.2 ± 230	668.5 ± 168	795.6 ± 69 ¥
TTP (ms)	51.7 ± 15.5 *	47.1 ± 10.9 *	45.1 ± 7.9 *	46.0 ± 6.9	38.4 ± 9.1	38.6 ± 4.0
½ RT (ms)	102.9 ± 66.8 **	68.5 ± 14.6 **#	59.2 ± 12.7 **#	56.7 ± 8.1	45.8 ± 9.6 #	46.5 ± 4.5 #

Note: *n* = number of animals in each group, MM muscle mass, CSA, cross-sectional area, MM:BM muscle mass to body mass ratio, Lo optimal length, Pt peak twitch force, TTP time to peak tension, ½ RT half relaxation time. Values are mean ± SD. * (*p* < 0.05), ** (*p* < 0.01), *** (*p* < 0.001) significantly different from contralateral values. # (*p* < 0.01) significantly different from control muscles at day 7 and/or day 14 post-injury. ¥ (*p* < 0.01) significantly different from WP-supplemented muscles at day 7 and/or day 14 post-injury.

**Table 3:** Muscle protein content of uninjured and injured EDL muscles from rats treated with standard rat chow- (CON), whey protein- (WP), or creatine- (CR) supplemented rat chow for 7 and 14 days.

Treatment	CON-INJ	WP-INJ	CR-INJ	CON-NORM	WP-NORM	CR-NORM
Duration						
**7 days**						
*n*	8	8	7	8	8	7
protein (mg/g)	116.3 ± 33.8 *	137.5 ± 38.5 *	142.9 ± 47.1 *	170.0 ± 33.8	163.8 ± 70.3	161.4 ± 43.7
contractile protein (mg/g)	30.6 ± 10.3 **	32.9 ± 10.7 **	41.9 ± 13.6 **	46.9 ± 17.2	43.8 ± 12.4	52.4 ± 8.6
% Contractile protein	28.3 ± 11.7	24.0 ± 4.5	30.7 ± 9.4	29.4 ± 12.9	32.5 ± 19.3	34.4 ± 10.2
**14 days**						
*n*	7	8	8	7	8	8
protein (mg/g)	127.1 ± 40.3 *	127.5 ± 41.3 *	156.3 ± 29.7 *	167.1 ± 35.9	145.0 ± 36.7	170.0 ± 20.0
contractile protein (mg/g)	32.4 ± 13.0	38.1 ± 12.8	46.6 ± 15.3 #¥	42.1 ± 11.4	36.3 ± 9.9	54.3 ± 14.1 #¥
% Contractile protein	25.3 ± 4.8	31.7 ± 10.7	29.3 ± 5.8	25.1 ± 5.9	25.9 ± 8.8	32.5 ± 9.7

Note: *n* = number of animals in each group. Values are mean ± SD. * (*p* < 0.05), ** (*p* < 0.01) significantly different from contralateral values. ¥ (*p* < 0.05) significantly different from WP-supplemented muscles at day 7 and/or day 14 post-injury.
